# Supplementary material for: Arabidopsis BTB-A2s Play a Key Role in Drought Stress
Source: Biology (Basel). 2024 Jul 26;13(8):561. doi: 10.3390/biology13080561 (PMC11351226; doi:10.3390/biology13080561)
Supplement: Supplementary file 1 [file biology-13-00561-s001.zip › Supplement Material.pdf]

Supplementary file

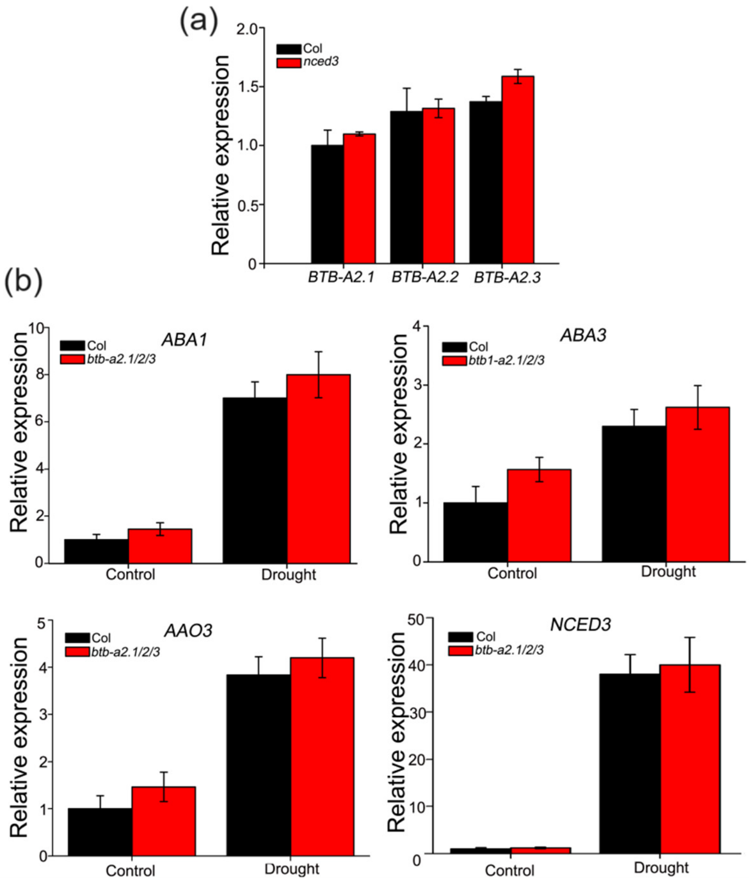

Figure S1. (**a**) Expression levels of *AtBTB-A2.1*, *AtBTB-A2.1*, and *AtBTB-A2.3* in *atnced3* mutant. (**b**) Expression levels of ABA synthesis relative genes in WT and triple mutant *btb-a2.1/2/3*. Total RNA was isolated from 7-day-old wild-type and *btb-a2.1/2/3* seedlings growing under normal and drought conditions. *AtACTIN2* gene was used as internal reference, and the results were shown by mean standard deviation. Data are mean ±SD. n=3.

Table S1. Analysis of *cis*-acting elements in *AtBTB-A2.1* promoter sequence

Name of the Element	Sequence	Function of the Element
DRE2COREZMRAB17	ACCGAC	*cis*-acting element involved in drought inducibility
DRECRTCOREAT	RCCGAC	*cis*-acting element involved in drought inducibility
MYCCONSENSUSAT	CANNTG	*cis*-acting element involved in drought and ABA responsiveness
RYREPEATBNNAPA	CATGCA	*cis*-acting element involved in seed specific gene expression
DPBFCOREDCDC3	ACACNNG	*cis*-acting element involved in ABA responsiveness
SURECOREATSULTR11	GAGAC	*cis*-acting element involved in sulfur responsiveness
MYB1AT	WAACCA	*cis*-acting element involved in drought and ABA responsiveness
MYB2CONSENSUSAT	YAACKG	dehydration-responsive element
MYBCORE	CNGTTR	dehydration-responsive element
REBETALGLHCB21	GAGAC	photoresponsive element
POLLEN1LELAT52	AGAAA	*cis*-acting element involved in pollen specific expression

Table S2. Analysis of cis-acting elements in *AtBTB-A2.2* promoter sequence

Name of the Element	Sequence	Function of the Element
ACGTATERD1	ACGT	*cis*-acting element involved in dehydration and etiolation
ABRELATERD1	ACGTG	abscisic acid responsiveness
DOFCOREZM	AAAG	Dof protein binding site, in endosperm
WRKY71OS	TGAC	pathogenesis, wound-induced response elements
SURECOREATSULTR11	GAGAC	*cis*-acting element involved in sulfur responsiveness
MYB1AT	WAACCA	*cis*-acting element involved in drought and ABA responsiveness
MYB2CONSENSUSAT	YAACKG	dehydration-responsive element
MYBCORE	CNGTTR	dehydration-responsive element
POLLEN1LELAT52	AGAAA	*cis*-acting element involved in pollen specific expression
WBOXATNPR1	TTGAC	pathogenesis-induced response elements
WBOXNTERF3	TGACY	wound-induced response elements
MYCCONSENSUSAT	CANNTG	drought and ABA responsiveness
GT1CONSENSUS	GRWAAW	SA regulatory elements

Table S3. Analysis of *cis*-acting elements in *AtBTB-A2.3* promoter sequence

Name of the Element	Sequence	Function of the Element
ABREOSRAB21	ACGTSSSC	abscisic acid responsiveness
ABRELATERD1	ACGTG	abscisic acid responsiveness
DOFCOREZM	AAAG	Dof protein binding site in endosperm
WRKY71OS	TGAC	pathogenesis, wound-induced response elements
DRECRTCOREAT	RCCGAC	*cis*-acting element involved in drought inducibility
MYB1AT	WAACCA	*cis*-acting element involved in drought and ABA responsiveness
MYB2CONSENSUSAT	YAACKG	dehydration-responsive element
MYBCORE	CNGTTR	dehydration-responsive element
POLLEN1LELAT52	AGAAA	*cis*-acting element involved in pollen specific expression
WBOXATNPR1	TTGAC	pathogenesis-induced response elements
WBOXNTERF3	TGACY	wound-induced response elements
MYCCONSENSUSAT	CANNTG	drought and ABA responsiveness
GT1CONSENSUS	GRWAAW	SA regulatory elements
ROOTMOTIFTAPOX1	ATATT	root specific expression element
REALPHALGLHCB21	AACCAA	photoresponsive element
DRE1COREZMRAB17	ACCGAGA	*cis*-acting element involved in drought inducibility
DPBFCOREDCDC3	ACACNNG	abscisic acid responsiveness

R represents A or G. W represents A or T. S represents C or G. Y represents C or T. K represents G or T. N represents G or A or T or C.

Table S4. Primers used in this study

Primer Name	Primer sequences (5'-3')
35S	GACGCACAATCCCACTATCC
BTB-A2.1-OE-F	ACGGGGGACTCTTGACCATGGATGAATTTTCCGACGATCC
BTB-A2.1-OE-R	GTCACCTGTAATTCACACGTGGTGGTGGTGGTGGTGCTCGAC GGATACCACACCGG
BTB-A2.2-OE-F	ACGGGGGACTCTTGACCATGGATGGTGGTTTCCGATGGCG
BTB-A2.2-OE-R	GTCACCTGTAATTCACACGTGGTGGTGGTGGTGGTGCTCGAC GGATACCACACCGG
BTB-A2.3-OE-F	ACGGGGGACTCTTGACCATGGATGGGTATCTCAAAAGACAG
BTB-A2.3-OE-R	GTCACCTGTAATTCACACGTGGTGGTGGTGGTGGTGTATGAT CGGACAAGGCGGAGT
BTB-A2.1-GUS-F	GCTCTAGATTCGCCAAGCAAACGCAG
BTB-A2.1-GUS-R	CGGGATCCGTTCGATTCATGAGGGATCGTC
BTB-A2.2-GUS-F	TGCCTGCAGGTCGACTCTAGACAACTATATGAAAGTAAGCG
BTB-A2.2-GUS-R	ATAAGGGACTGACCACCCGGGGACACTCGTTTGCCGCCATC
BTB-A2.3-GUS-F	GCTCTAGAGACCTTGAGTCCTTGACGAT
BTB-A2.3-GUS-R	CGGGATCCTCCACCAACGTTGAATTTGAT
BTB-A2.1-qRT-F	AGGGGAAGTTTTCTACGCCG
BTB-A2.1-qRT-R	GCTTGCATTCCCCACCAAAC
BTB-A2.2-qRT-F	GGAACCGTCCGTACACATCT
BTB-A2.2-qRT-R	TTCCGAATCAGCAACGGCG
BTB-A2.3-qRT-F	GCCAACATCCCTGAGCGTCT
BTB-A2.3-qRT-R	ATCAGGACCTGCCCTGATGGC
ABI5-qRT-F	ATGATCAAGAACCGCGAGTCTGC
ABI5-qRT-R	CGGTTGTGCCCTTGACTTCAAAC
RAB18-qRT-F	GGCTTGGGAGGAATGCTTCA
RAB18-qRT-R	CGCTTGAGCTTGACCAGACT
RD29A-qRT-F	GGAAGTGAAAGGAGGAGGAGGAA
RD29A-qRT-R	CACCACCAAACCAGCCAGATG
RD29B-qRT-F	GAATCAAAAGCTGGGATGGA
RD29B-qRT-R	TGCTCTGTGTAGGTGCTTGG
ABA1-qRT-F	CGTGCGGTTGGAGAAGATGTGAT
ABA1-qRT -R	TCTCAGAATGGCTTCCTCCTCAGT
ABA3-qPCR-F	AGTGGATATTGAAGAGGCAGC
ABA3-qPCR-R	CACCAGATCTAGATTAAACCTCAGG
AAO3-qRT -F	CAACCGCATGCGCACTAG
AAO3-qRT -R	GTCTTGCGGTTCAAAAACATCTT
NCED3-qPCR-F	GAGTGTCCTGTCTGAAATCCG
NCED3-qPCR-R	CGAATCCTGAGACTTTAGGCC
ACTIN2-F	ACTCTCCCGCTATGTATGTCGCC
ACTIN2-R	ATTTCCCGCTCTGCTGTTGTGGT
